# Association of variants m.T16172C and m.T16519C in whole mtDNA sequences with high altitude pulmonary edema in Han Chinese lowlanders

**DOI:** 10.1186/s12890-021-01791-1

**Published:** 2022-02-25

**Authors:** Yan Wang, Xuewen Huang, Fujun Peng, Huiling Han, Yanan Gu, Xin Liu, Zhichun Feng

**Affiliations:** 1 Clinical Biobank Center, Medical Innovation Research Division of Chinese, PLA General Hospital, No. 28 Fu Xin Road, Hai Dian District, Beijing, 100853 China; 2 BaYi Children’s Hospital, The Seventh Medical Center of PLA General Hospital, No.5 Nan Men Cang, Dong Cheng District, Beijing, 100700 China; 3 The Mountain Sickness Prevention Research Center of the General Hospital of Tibet Military Command, Tibet, China; 4 School of Basic Medical Sciences, Weifang Medical University, Weifang, Shandong China

**Keywords:** Genotype, Haplogroups, Han Chinese, High altitude pulmonary edema, Whole mtDNA sequences

## Abstract

**Background:**

High altitude pulmonary edema (HAPE) is a hypoxia-induced non-cardiogenic pulmonary edema that typically occurred in un-acclimatized lowlanders, which inevitably leads to life-threatening consequences. Apart from multiple factors involved, the genetic factors also play an important role in the pathogenesis of HAPE. So far, researchers have put more energy into the nuclear genome and HAPE, and ignored the relationship between the mitochondrion DNA (mtDNA) variants and HAPE susceptibility.

**Methods:**

We recruited a total of 366 individuals including 181 HAPE patients and 185 non-HAPE populations through two times. The first time, 49 HAPE patients and 58 non-HAPE individuals were performed through whole mtDNA sequences to search the mutations and haplogroups. The second time, 132 HAPE patients and 127 non-HAPE subjects were collected to apply verifying these mutations and haplogroups of mtDNA with the routine PCR method.

**Results:**

We analyzed and summarized the clinical characteristics and sequence data for the 49 HAPE patients and 58 non-HAPE individuals. We found that a series of routine blood indexes including systolic arterial blood pressure (SBP), heart rate (HR), white blood cell (WBC), and C-reactive protein (CRP) in the HAPE group presented higher and displayed significant differences compared with those in the non-HAPE group. Although the average numbers of variants in different region and group samples were not statistically significant (*P* > 0.05), the mutation densities of different regions in the internal group showed significant differences. Then we found two mutations (T16172C and T16519C) associated with the HAPE susceptibility, the T16172C mutation increased the risk of HAPE, and the T16519C mutation decreased the HAPE rating. Furthermore, the two mutations were demonstrated with 132 HAPE patients and 127 non-HAPE individuals. Unfortunately, all the haplogroups were not associated with the HAPE haplogroups.

**Conclusions:**

We provided evidence of differences in mtDNA polymorphism frequencies between HAPE and non-HAPE Han Chinese. Genotypes of mtDNA 16172C and 16519C were correlated with HAPE susceptibility, indicating the role of the mitochondrial genome in the pathogenesis of HAPE.

**Supplementary Information:**

The online version contains supplementary material available at 10.1186/s12890-021-01791-1.

## Background

High altitude pulmonary edema (HAPE) is an acute idiopathic mountain disease that usually occurs quickly in lowlanders when they are exposed to an altitude exceeding 2500 m above sea level. HAPE is considered a life-threatening non-cardiogenic disease [1]. In some cases, HAPE can develop at the altitude of 1500 - 2500 m for highly susceptible populations [2]. This disease typically occurs 2 - 5 days after arriving at the altitude and is associated with insidiousness [3]. The mortality rate of untreated HAPE is up to 50%, while the mortality rate of treated HAPE is decreased to 11% [3]. Early symptoms of HAPE include non-productive cough, exertional dyspnea, chest pain, and reduced exercise tolerance. Without treatment, HAPE can progress to dyspnea at rest [3]. This disease is caused by a variety of risk factors including hypoxic ventilatory response, rapidly ascent, high altitude, tired, lacking sufficient sleeping, smoking, drinking, heredity, etc. [3–6]. In addition, the patients with basic disease such as hypertension and diabetes are easier to develop HAPE [7, 8]. Therefore, when studying the relationship between genetics and HAPE, the risk factors should be eliminated.

So far, the major pathogenic mechanism of HAPE is the imbalance and mutations of the pulmonary endothelial cells (ECs) and vascular smooth muscle cells (VSMCs), which leads to the accumulation of extravascular fluid in the lungs and then lung malfunction [9, 10]. A large number of studies have found that many pathways participate in the development of HAPE, such as renin–angiotensin–aldosterone system (RAAS) [11], hypoxia-inducible factor (HIF) pathway [12], the nitric oxide (NO) pathway [13], the mitochondrion DNA (mtDNA) [14, 15], *etc*. For HAPE patients, suitable treatments include oxygen suction and lowland transferring, which suggests that HAPE is very sensitive to the changes in oxygen/energy metabolism [15–17]. Mitochondria are major ATP energy production centers in eukaryotic cells, and their function can be affected by the mtDNA variants [18]. Therefore, the relationship between HAPE and mtDNA is important for understanding the pathogenic mechanism of HAPE.

MtDNA is an extranuclear double-stranded DNA found only in mitochondria. In most eukaryotes, mtDNA is a circular molecule and is inherited from the mother. The full length of mtDNA is 16,569 base pairs (bp), divided into heavy (H) brand and light (L) brand, including 37 genes encoding 2 rRNA (12S rRNA and 16S rRNA), 22 tRNA, and 13 polypeptide subunits (Complex I, III, IV, and V) that produce mitochondrial ATP and participate in the oxidative phosphorylation together with proteins coded by nuclear genome [19]. In addition to coding region, mtDNA also contains a part of the non-coding region called control region or displacement loop (D-loop), which can be divided into high variant sequencing-I (HVS-I) and high variant sequencing-II (HVS-II), and is vital to mtDNA replication and regulation [20]. Mitochondrial function can be affected by variations in mtDNA, including polymorphisms, content changes, and deletions [21]. These variants play an important role in acclimatizing or adapting to hypoxia [21]. In this study, we mainly focused on the association between mtDNA mutants and HAPE susceptibility. For example, Luo et al. used polymerase chain reaction (PCR) methods instead of whole mtDNA sequences to reveal that genotypes of mtDNA 3397G and 3552A were correlated with HAPE susceptibility in Han Chinese [22]. Some results suggested that mitochondrial haplogroups B and M7 were associated with the inadaptability of hypoxic environments, whereas haplogroups G and M9a1a1c1b may be associated with hypoxic adaptation [23]. Recently, literature presented that variants G4491A, A4944G, and A14002G associated with haplogroup M33a2′3 may be the main reasons for the susceptibility of Indian male lowlanders to HAPE, however, the study only included 15 HAPE patients and 20 non-HAPE individuals through long PCR and the results were not verified [15].

Even though these results have already established the association between mtDNA mutations and HAPE, there are still some problems worth exploring and studying, such as limited samples, verification of polymorphisms and haplogroups. In this study, we recruited two groups of people. The first group including 51 HAPE patients (2 patients were removed due to poor quality of mtDNA) and 58 non-HAPE persons was studied using whole mtDNA sequences to find the possible association between mutations and haplogroups. The second group including 132 HAPE patients and 127 non-HAPE patients was used to verify some variants of mtDNA with the routine PCR method. We found two mutations (T16172C and T16519C) associated with the HAPE susceptibility using the largest samples so far. The T16172C mutation indicated an increased susceptibility to HAPE, and the T16519C mutation could reduce the risk factor for obtaining HAPE.

## Methods

### Samples collections

In this study, a total of 366 subjects were recruited, including 181 HAPE patients and 185 non-HPAE subjects from September 2018 to September 2020, and divided into 2 groups. The first group contained 51 HAPE patients and 58 non-HAPE people who were low-altitude Han-Chinese and climbed rapidly to 3658 m in Lhasa, Tibet, China within 7 days (Table 1 and Additional file 1). In addition, the first group of subjects were male and met the following conditions: (1) HAPE patients were diagnosed according to the clinical standard requirements, which mainly included cough, dyspnea at rest, white or pink foamy sputum, central cyanosis, pulmonary crackles, and the presence of flake or cloud infiltrate shadows at unilateral or bilateral pulmonary hilar on chest X-ray [24, 25]. (2) They were from the General Hospital of Tibetan Military Command and had no relationship with each other. (3) They did not have a history of smoking, drinking, hypertension, diabetes, cardiopulmonary disease, acute or chronic pulmonary infection (e.g., pneumonia), and other mtDNA-related diseases. (4) The non-HAPE had no acute mountain sickness after moving to Lhasa for 7 days. The second group had 132 HAPE patients and 127 non-HAPE individuals, and also met the above screening criteria (Additional file 1).

**Table 1:** Basic clinical characteristics of all the subjects

	HAPE (n = 49)	non-HAPE (n = 58)	*P*-value
Gender	Male	Male	
Race	Han	Han	
Smoking	No	No	
Drinking	No	No	
Age	27.6 ± 6.7	25.4 ± 5.9	0.082
SBP (mmHg, < 140)	127.0 ± 19.7	118.5 ± 11.3	< 0.0001**
DBP (mmHg, < 90)	85.2 ± 14.5	80.7 ± 10.4	< 0.0001**
HR (bpm, 60–100)	106.1 ± 17.5	90.7 ± 14.4	< 0.0001**
WBC (× 10^9^/L, 3.5–9.5)	11.0 ± 3.7	8.1 ± 3.6	< 0.0001**
RBC (× 1012/L, 4.4–6.0)	5.4 ± 0.7	5.4 ± 0.5	0.073
HCT (%, 49–57)	48.2 ± 4.7	50.3 ± 4.3	< 0.0001**
Hb (g/L, 144–175)	157.0 ± 16.7	168.3 ± 10.1	< 0.0001**
CRP (mg/L, 0–10)	50.9 ± 37.4	37.6 ± 34.6	< 0.0001**

SBP, systolic arterial blood pressure; DBP, diastolic arterial blood pressure; HR, heart rate; WBC, white blood cell; RBC, red blood cell; HCT, hematocrit; Hb, hemoglobin concentration; CRP, C-reactive protein

Data are presented as mean ± SD. **¶**, t-test; *, *P*-value < 0.05; **, *P*-value < 0.01

### DNA extraction, sequencing, and haplogroup classification

Blood samples were collected from all the subjects and stored at − 80 ℃. Genomic DNA was extracted from peripheral blood using Magbead Blood DNA Kit (CWBio, Beijing, China). For each patient, 200 ng genomic DNA was sheared by Biorupter (Diagenode, Belgium) to acquire 150 - 200 bp fragments. The ends of the DNA fragment were repaired, and Illumina Adaptor was added (Fast Library Prep Kit, iGeneTech, Beijing, China). After the sequencing library was constructed, the whole exons were captured with AI-Mito-Cap (iGeneTech, Beijing, China) and sequenced on the Illumina platform (Illumina, San Diego, CA), with 150 base paired‐end reads. Raw reads were filtered by FastQC to remove low-quality reads. Then clean reads were mapped to the reference genome GRCh37 using Bwa. After duplications were removed, SNV and InDel were called and annotated by GATK, SamtoolS, Varscan. According to the PhyloTree Build 17 standard, using the software development tool MitoTool, the haplogroups of entire mtDNA sequences were constructed using the phylogenetic analysis method [26–28]. When evaluating the mtDNA variants, a series of affecters were considered, including the different variants of the particular branches of each haplogroup, the calescence time of variation distribution, and the location of protein- or RNA-encoded gene-based substitutions. All these factors were integrated into phylogenetic analysis to construct Haplogroup.

### PCR conditions

We directly sequenced the PCR products to genotype the A263G, T310N, 310-311insC, T16172C, and T16519C polymorphisms in the remaining individuals. The primers (forward: 5’-CAGCCACCATGAATATTGTACG-3’, reverse: 5’-GTTAGGCTGGTGTTAGGGTTC-3’) were designed using the Primer 5.0 software (PREMIER Biosoft International, Palo Alto, CA, USA). The reactions were performed in 50 μL volume containing 50 ng template DNA, 5 mM dNTP (Takara, Dalian, China), 10 pmol forward primers, 10 pmol reverse primers, 2.5 U Taq DNA polymerase enzyme (Takara) in Taq buffer, and 75 mM MgCl_2_. Then PCR amplification was performed under the following conditions: a pre-denaturation cycle at 94 °C for 4 min; 45 cycles at 94 °C for 20 s, 56 °C for 30 s, 72 °C for 1 min; and a final extension cycle at 72 °C for 10 min. The obtained products were cooled to 4 °C. The PCR products were directly sequenced by Shanghai Sangon Biological Engineering Technology & Services Co., Ltd (Shanghai, China). The sequenced results were assigned with the standard mitochondrial reference genome called Revised Cambridge reference sequence (rCRS). To verify the variants A3397G and T3552A, we used the primers (forward: 5’- GTCCTACGTGATCTGAGTTCAG-3’, reverse: 5’-GCTAGGGTGACTTCATATGAG-3’).

### Ethical statement

All the samples of this study collected had written informed content from patients. All clinical and experimental steps were approved by the Ethical Committee of the Seventh Medical Center of PLA General Hospital and the Ethical Committee of the General Hospital of Tibetan Military Command. Meanwhile, these contents were conducted in accordance with the Helsinki Declaration guideline.

### Statistical analyses

All the statistical analyses of data were performed using SPSS 17.0. Pearson χ2 test was used to assess the significance of differences in haplogroup and SNP frequencies between HAPE and non-HAPE groups. Quantitative data were expressed as mean ± standard deviation (mean ± SD) and the unpaired student’s t-tests were used to compare the HAPE group and non-HAPE group. The *P*-values, odds ratios (ORs), and 95% confidence intervals (95% CI) were calculated. A two-sided *P*-value < 0.05 was considered statistically significant. All P-values were adjusted by Bonferroni correction.

## Results

### Clinical characteristics of all the individuals

Firstly, in the first group, 2 HAPE patients were removed because of the poor-quality DNA levels. Then, a total of 49 HAPE patients and 58 non-HAPE individuals were further analyzed. They had same screening conditions and suffered similar environments. Their detailed clinical information is listed in Table 1 and Additional file 1.

As shown in Table 1, the age distribution was consistent between the HAPE patients and non-HAPE individuals. Except for red blood cell (RBC), all the routine blood indexes such as systolic arterial blood pressure (SBP), white blood cell (WBC), and C-reactive protein (CRP) in HAPE patients were significantly different from non-HAPE (*P* < 0.05), which was probably related to the adaption of high environments [1, 29, 30].

### Data summary of sequencing and quality control

**Fig. 1:**
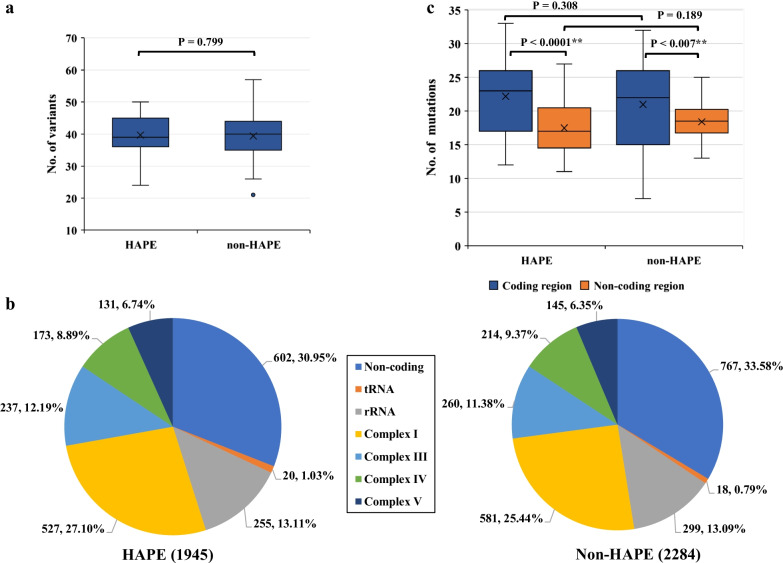
Distribution of variants in HAPE and non-HAPE groups. **a** Number of variants per sample. **b** Number of variants in coding regions and non-coding regions in per sample. **c** Number and proportion of mutations in different regions. Left, HAPE group; Right, non-HAPE group. *P*-value was obtained in t-test; *, *P*-value < 0.05; **, *P*-value < 0.01

**Fig. 2:**
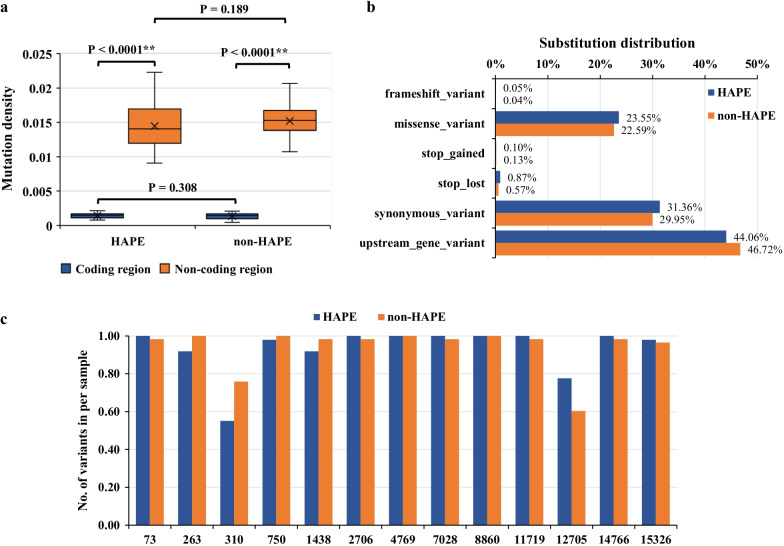
Gene distributions in mitochondrial genome of HAPE and non-HAPE groups. **a** Mutation densities (mutation number in a region/region bp length) in coding regions and non-coding regions of HAPE or non-HAPE group. **b** Number and proportion of frameshift variants, missense variants, stop gained, stop lost, synonymous variants and upstream gene variants. **c** Mutation rates in HAPE or on-HAPE individuals. *P*-value was obtained in t-test; *, *P*-value < 0.05; **, *P*-value < 0.01

The mtDNA of the first group samples were isolated and sequenced using the Illumina platform. Nearly 92% of total data generated a Q > 30 Phred score. In addition, after removing the duplications, the mean sequence depth of 8400 × was observed, which accounted for 24.6% on average. The whole mitochondrial genome region was covered for all the samples. All the variants of HAPE patients and non-HAPE individuals are listed in Additional file 2 and Additional file 3. After processing these variants, the detailed and summarized results are shown in Figs. 1 and 2, respectively.

Compared to the non-HAPE group, the mean numbers of variants in HAPE patients were higher; however, the difference between non-HAPE and HAPE groups was not significant (*P* = 0.799) (Fig. 1a). In HAPE group, a total of 1945 mutations were distributed in non-coding region (602, 30.95%), tRNA region (20, 1.03%), rRNA region (255, 13.11%), complex I (527, 27.10%), complex III (237, 12.19%), complex IV (173, 8.89%), and complex V (131, 6.74%) (Fig. 1b). There were 2284 mutations in non-HAPE individuals, which were located in non-coding region (767, 33.58%), tRNA region (18, 0.79%), rRNA region (299, 13.09%), complex I (581, 25.44%), complex III (260, 11.38%), complex IV (214, 9.37%), and complex V (145, 6.35%) (Fig. 1b). Unfortunately, the two randomly corresponding regions did not show significant differences (Additional file 4). Then, we analyzed the numbers of variants of coding and non-coding regions in HAPE and non-HAPE groups. The results indicated that the differences between HAPE and non-HAPE groups were not significant (Fig. 1c). However, the average variants between the coding region and the non-coding region in HAPE patients were significantly different, which may be related to the length of coding regions and non-coding regions in the sequence. Similar findings were found in the non-HAPE group (Fig. 1c).

To further explore the variants’ distributions characteristics of the mitochondrial genome in HAPE and non-HAPE patients, we used the mutation densities (mutation number in a region/region bp length) to describe the variants distributions in coding regions and non-coding regions. The results indicated that the mutation densities in coding regions or non-coding regions were consistent between the HAPE and non-HAPE groups (Fig. 2a). However, within the same group (HAPE or non-HAPE group), the mutation densities in the non-coding regions were higher than those in the coding regions (Fig. 2a). This suggested the sequences of non-coding regions were inclined to mutations. In all the variants, the top three molecular consequences in both the HAPE group and non-HAPE group were 44.06% upstream gene, 31.36% synonymous, and 23.55% missense variants (Fig. 2b), and there was no significant difference between the HAPE group and non-HAPE group (Additional file 5). Interestingly, a few positions in the mitochondrial genome had very high mutation rates (variants number/samples number), such as 73rd position (1.0), 2706th position (1.0), 8860th position (1.0) in HAPE patients (Fig. 2c and Additional file 6). Meanwhile, these positions in the non-HAPE group also showed similar results and the difference between HAPE and non-HAPE groups was not significant (Fig. 2c and Additional file 6). The results suggested that the mitochondrial genome had some high-frequency mutation sites that were not associated with HAPE.

### Association of mtDNA variants with HAPE susceptibility

In this study, a total of 1945 mutations and 2284 mutations were observed in HAPE and non-HAPE groups, respectively, which were located in 483 variants’ sites and 530 variants’ sites (combined with 742 variants’ sites), respectively (Additional file 6). There were 18 different and typical mutations in Table 2, including 5 variants sites (A263G, T310N, (310–311) insC, T16172C, and T16519C) with significant differences between both groups, 6 variants sites (A8459G, C8684T, C14067T, T14470C, and A16164G) only found in HAPE group, 3 variants sites (C194T, C6960T, and G16390A) only found in the non-HAPE group, and 4 variants sites (T11944C, A15235G, C16291T, and A16316G) had high odds ratio (OR) values (Table 2). After Bonferroni correction, only 4 mutations (T310N, (310–311) insC, T16172C, and T16519C) were statistically significant between HAPE and non-HAPE groups (P < 0.05), and they were located in the D-loop of mtDNA non-coding regions. Since the 310 site was short repeats sequences C bases, this site was removed in further analysis. Our results showed that the frequencies of T16172C in the HAPE group (20.41%) were higher than those in the non-HAPE group (5.17%, *p*-adjust = 0.035, OR = 4.701, and 95%CI = 1.214–18.204). In contrast, the frequencies of T16519C in HAPE patients (34.69%) were significantly lower than those in non-HAPE individuals (67.24%, *p*-adjust = 0.002, OR = 0.259, and 95%CI = 0.116–0.578) (Table 2). The two mutations were previously reported in other studies, but not in high-altitude diseases [31–33]. Then, we also statistically analyzed the number of T16172C mutation and T16519C mutation existing in a certain body at the same time. there was no difference between HAPE patients and non-HAPE individuals (Additional file 7).

**Table 2:** Comparison of variant frequencies between HAPE and non-HAPE groups

Site	Genotype	Gene	Mutation	Protein change	HAPE (n = 49)	non-HAPE (n = 58)	HAPE versus non-HAPE			
							*P*-value	*P*-adjust	OR	95%CI
C194T	T	TRNF	c.-383C > T		0 (0%)	3 (5.17%)	0.106	0.304	1.055	0.993–1.120
A263G	G	TRNF	c.-314A > G		45 (91.84%)	58 (100%)	0.027*	0.088	0.918	0.845–0.998
T310N	N	TRNF	c.-267T > N		27 (55.10%)	44 (75.86%)	0.024*	0.039*	0.390	0.171–0.890
(310–311) insC	insC	TRNF	c.-267_-266insC		22 (44.90%)	14 (24.14%)	0.024*	0.039*	2.561	1.124–5.836
G6179A	A	COX1	c.276G > A	p. Met92Ile	3 (6.12%)	0 (0%)	0.056	0.186	0.939	0.874–1.008
C6960T	T	COX1	c.1057C > T	p. Leu353Leu	0 (0%)	3 (5.17%)	0.106	0.304	1.055	0.993–1.120
A8459G	G	ATP8	c.94A > G	p. Asn32Asp	3 (6.12%)	0 (0%)	0.056	0.186	0.939	0.874–1.008
C8684T	T	ATP6	c.158C > T	p. Thr53Ile	3 (6.12%)	0 (0%)	0.056	0.186	0.939	0.874–1.008
T11944C	C	ND4	c.1185T > C	p.Leu395Leu	4 (8.16%)	1 (1.72%)	0.116	0.266	5.067	0.547–46.926
C14067T	T	ND5	c.1731C > T	p. Thr577Thr	3 (6.12%)	0 (0%)	0.056	0.186	0.939	0.874–1.008
T14470C	C	ND6	c.204A > G	p. Gly68Gly	3 (6.12%)	0 (0%)	0.056	0.186	0.939	0.874–1.008
A15235G	G	CYTB	c.489A > G	p.Ter163Trpext*?	1 (2.04%)	4 (6.90%)	0.236	0.468	0.281	0.030–2.604
A16164G	G	ND6	c.-1491T > C		3 (6.12%)	0 (0%)	0.056	0.186	0.939	0.874–1.008
T16172C	C	ND6	c.-1499A > G		10 (20.41%)	3 (5.17%)	0.016*	0.035*	4.701	1.214–18.204
C16291T	T	ND6	c.-1618G > A		4 (8.16%)	1 (1.72%)	0.116	0.266	5.067	0.547–46.926
A16316G	G	ND6	c.-1643T > C		4 (8.16%)	1 (1.72%)	0.116	0.266	5.067	0.547–46.926
G16390A	A	ND6	c.-1717C > T		0 (0%)	3 (5.17%)	0.106	0.304	1.055	0.993–1.120
T16519C	C	ND6	c.-1846A > G		17 (34.69%)	39 (67.24%)	0.001**	0.002**	0.259	0.116–0.578

*P*-value, Person test; *P*-adjust, adjust Person’s value; OR, odds ratio; CI, confidence interval. *, *P*-value < 0.05; **, *P*-value < 0.01

### Haplogroup comparison between HAPE and non-HAPE patients

A total of 107 complete mtDNA sequences of the first group’s patients were analyzed using MitoTool software according to the PhyloTree Build 17 criteria (GenBank J01415.2) [26–28]. We found that all the mtDNA sequences were mapped to the single initial haplogroup L3, which originated from Africa and was composed of two macrohaplogroups, M and N (Table 3). All the subjects belonged to 16 haplogroups. No differences were observed in the haplogroups between the HAPE and non-HAPE groups (*P* > 0.05, Table 3). These haplogroups were further divided into the next-level haplogroups, which were not statistically significant (*P* > 0.05, Additional file 8).

**Table 3:** Distribution of haplogroups in HAPE and non-HAPE groups

Macrohaplogroup	Haplogroup	HAPE		Non-HAPE		HAPE versus non-HAPE			
		(n = 49)	%	(n = 58)	%	*P*-value	*P*-adjust	OR	95% CI
M	C	3	6.82%	3	5.17%	0.831	1.000	1.196	0.230–6.210
	D	12	27.27%	10	17.24%	0.355	0.494	1.557	0.607–3.995
	G	4	9.09%	2	3.45%	0.291	0.526	2.489	0.436–14.210
	M21	0	0.00%	1	1.72%	0.356	1.000	1.018	0.983–1.053
	M7	5	11.36%	11	18.97%	0.205	0.320	0.486	0.156–1.509
	M8a	3	6.82%	0	0.00%	0.056	0.186	0.939	0.874–1.008
	M9	2	4.55%	1	1.72%	0.462	0.882	2.426	0.213–27.588
	Z	0	0.00%	1	1.72%	0.356	1.000	1.018	0.983–1.053
N	A	5	11.36%	3	5.17%	0.324	0.537	2.083	0.472–9.200
	B	7	15.91%	15	25.86%	0.140	0.216	0.478	0.177–1.289
	F	4	9.09%	5	8.62%	0.932	1.000	0.942	0.239–3.721
	H	0	0.00%	1	1.72%	0.356	1.000	1.018	0.983–1.053
	N9a	2	4.55%	2	3.45%	0.863	1.000	1.191	0.162–8.786
	R11	0	0.00%	1	1.72%	0.356	1.000	1.018	0.983–1.053
	R9b	0	0.00%	1	1.72%	0.356	1.000	1.018	0.983–1.053
	Y	1	2.27%	0	0.00%	0.274	0.932	0.980	0.941–1.020

*P*-value, Person test; P-adjust, adjust Person’s value; OR, odds ratio; CI, confidence interval

### Validation of two mutations (T16172C and T16519C)

In order to validate the association between two mutations (T16172C and T16519C) and HAPE susceptibility, the subjects of the second group were collected (Additional file 1). Their basic clinical characteristics were listed in Additional file 9. The PCR results of these variants are shown in Table 4. The frequencies of T16172C in HAPE patients (44, 33.33%) were higher than those in non-HAPE individuals (27, 21.26%, *p*-adjust = 0.042, OR = 1.852, and 95%CI = 1.060–3.236). The frequencies of T16519C in HAPE group (52, 39.39%) were lower than the non-HAPE group (67, 52.76%, *p*-adjust = 0.042, OR = 0.582, and 95%CI = 0.355–0.953). The above results indicated that the T16172C mutation increased the HAPE susceptibility and the T16519C mutantation decreased the impact from HAPE. Meanwhile, we also analyzed the number of 16172C-16519C mutation between HAPE patients and non-HAPE individuals in the second group and all the subjects. Similar to the result of the first group, there was no difference (Additional file 7).

**Table 4:** Validation of association between mt16172C, mt16519C genotypes and HAPE susceptibility

Position	Genotype	HAPE (n = 132)	non-HAPE (n = 127)	*P*-value	*P*-adjust	OR	95% CI
16172	C	44 (33.33%)	27 (21.26%)	0.029	0.042	1.852	1.060–3.236
	T	88 (66.67%)	100 (78.74%)				
16519	C	52 (39.39%)	67 (52.76%)	0.031	0.042	0.582	0.355–0.953
	T	80 (60.61%)	60 (47.24%)				

*P*-value, Person test; *P*-adjust, adjust Person’s value; OR, odds ratio; CI, confidence interval

## Discussion

HAPE is a severe acute disease caused by high-altitude hypoxia. Several studies have found that the mutations of mtDNA associated with the HAPE susceptibility; however, these results can’t be verified by other samples [22, 34]. In this study, we found two mutations (T16172C, T16519C) located in the D-loop of the non-coding region were associated with HAPE susceptibility in Han Chinese, which were validated with PCR method in the other group individuals. The frequencies of T16172C mutation in the HAPE group were higher than those in the non-HAPE group. In contrast, the 16519C mutations tended to reduce the HAPE susceptibility. Two mutations were also reported previously in the other diseases, but not in high-altitude diseases [31–33].

Previous study had found that the 16172 (rs2853817) and 16519 (rs3937033) mutations showed preliminary association with mitochondrial function without changing primary sequences of protein [35]. The site of T16172C mutation was located in the hypervariable region of mtDNA. So far, little is known about the T16172C mutation in other diseases including mountain sickness, except for one paper that demonstrated the relationship between the T16172C and ataxia telangiectasia for twins [33]. The site of mtDNA 16519 has been reported to have the highest worldwide mutation rates [35], and has an association with some diseases. Navaglia et al. found that the T allele of mtDNA 16519 SNP was correlated with shorter life expectancy in pancreatic cancer [31]. The relationship between 16519 site and other cancers such as breast cancer and familial nasopharyngeal carcinoma has also been demonstrated [36, 37]. Liao et al. reported that the T16519C mutation showed a susceptible tendency to type-2 diabetes mellitus (T2DM) in the Chinese Han population [38]. Significant differences were also found in the change rate of VO (2 max) and citrate synthase activity as a result of training between the two groups at 16519 site [39]. Besides, this mutation might cause other common disorders such as migraine headaches or psychiatric disorders such as schizophrenia [32, 40, 41]. So far, the T16172C and T16519C variants in mtDNA were first reported to be associated with the HAPE of mountain sickness. Although both the 16172 and 16519 variants are located in the D-loop region of mtDNA, they may act by changing the transcription levels of mitochondrial proteins, which are related to oxidative phosphorylation, and the changes in this process may lead to β-cell failure or ATP production lack [42, 43]. These phenomena need to be further verified in future research.

About ten years ago, Luo et al. [22] found that genotypes of mtDNA 3397G and 3552A were correlated with HAPE susceptibility, which were not verified in this paper (Additional file 10). As we all know, HAPE is caused by caused by multiple factors including environmental and genetic reasons, such as hypoxic ventilatory response, rapid ascent, high altitude, tiredness, lacking sufficient sleeping, smoking, drinking, heredity, etc. [3–6]. In addition, the patients with basic diseases such as hypertension and diabetes are easier to develop HAPE [7, 8]. Thus, the different clinical samples could lead to different results.

Besides variant genotypes of mtDNA, the mitochondrial haplogroup classification in HAPE and non-HAPE individuals was performed using MitoTool. mtDNA is a well-known genetic marker due to the characteristics of high mutation rate, maternal inheritance, high copy number, and lack of recombination, which makes it different from the nuclear genome [34]. On this basis, mtDNA haplogroups such as the non-recombining region of Y-chromosome are usually considered as the main measures for investigating human evolution and origin [44, 45]. Mitochondrial DNA haplogroups are a characteristic cluster of tightly linked mtDNA polymorphism that forms continent-specific genotypes [44]. Studies have also shown that certain mitochondrial haplogroups are predisposed to high-altitude diseases while others can prevent mountain sickness. For example, the studies on the mitochondrial genome had revealed that there was a significant difference in the frequency of the mitochondrial 3010G-3970C haplotype between high- and low-altitude populations, which was believed to be associated with the improved adaptability of the Tibetan population to the low-oxygen environment [46]. Other studies had also shown that the mitochondrial haplogroups were associated with high-altitude adaptation, such as haplogroups B, M7, M9a1a1c1b, B4b, D4, etc. [23, 34, 47]. These mtDNA haplogroups were also observed in our samples; however, we found that they were not significantly different between HAPE and non-HAPE groups (Additional file 8). This phenomenon was inconsistent with the results in the previous study that mtDNA haplogroup M33a2′3 may be linked with the HAPE susceptibility of 15 HAPE patients and 20 non-HAPE individuals who were male and came from Indian [15].

Although we have found two mutations (T16172C, T16519C) associated with the HAPE susceptibility, this study still has limitations, including relatively small sample size and lack of functional experiments to identify the effects of the genetic variants on gene structure/function. Therefore, our findings need to be validated in a larger population and the functional significance of the important genetic variants should be further investigated.

## Conclusions

In this study, we established the associations between mtDNA mutations and HAPE susceptibility. The T16172C mutation increased the risk of HAPE, while the variant of T16519C tended to be resistant to HAPE. Then, these two mutations were verified with the other group of patients. We also found haplogroup of mtDNA may not play a role in the HAPE susceptibility. Meanwhile, we found that a few routine blood indexes, such as SBP, HR, WBC, and CRP, showed significant differences between the HAPE group and the non-HAPE group. So, these findings would enrich the association between mtDNA and HAPE and can provide a new perspective to reveal the genetic mechanism of HAPE.

## Supplementary Information

**Additional file 1**: Basic clinical information of all the patients.

**Additional file 2**: The mtDNA sequences results of all the HAPE patients.

**Additional file 3**: The mtDNA sequences results of all the non-HAPE persons.

**Additional file 4**: Number and proportion of mutations in different regions of mtDNA.

**Additional file 5**: Number and proportion of molecular consequence in mtDNA.

**Additional file 6**: Comparison of variant frequencies between HAPE and non-HAPE groups.

**Additional file 7**: Validation of association between 16172C-16519C genotypes and HAPE susceptibility.

**Additional file 8**: Distribution of haplogroups in HAPE and non-HAPE groups.

**Additional file 9**: Basic clinical characteristics of all the validating subjects.

**Additional file 10**: Validation of association between mt3397G, mt3552A genotypes and HAPE susceptibility.

## Data Availability

The data that support the findings of this study are available from the corresponding author upon reasonable request.

## Abbreviations

bp: Base pairs
CI: Confidence intervals
CRP: C-reactive protein
D-loop: Displacement loop
ECs: Endothelial cells
H: Heavy
HAPE: High altitude pulmonary edema
HIF: Hypoxia-inducible factor
HR: Heart rate
HVS-I: High variant sequencing-I
HVS-II: High variant sequencing-II
L: Light
mtDNA: Mitochondrion DNA
NO: Nitric oxide
ORs: Odds ratios
PCR: Polymerase chain reaction
RAAS: Renin angiotensin aldosterone system
RBC: Red blood cell
rCRS: Revised Cambridge reference sequence
SBP: Systolic arterial blood pressure
T2DM: Type-2 diabetes mellitus
VSMCs: Vascular smooth muscle cells
WBC: White blood cell
